# Registered nurse case managers’ work experiences with a person-centered collaborative healthcare model: an interview study

**DOI:** 10.1186/s12913-024-11500-3

**Published:** 2024-09-23

**Authors:** Markus Hjelm, Anna Andersson, Venera Ujkani, Ewa Kazimiera Andersson

**Affiliations:** 1 Blekinge Centre of Competence, Region Blekinge, Karlskrona, Sweden; 2 Department of Clinical Sciences in Malmö, Lund University, Lund, Sweden; 3 Department of Health and Caring Sciences, Linnaeus University, Växjö, Sweden

**Keywords:** Case management, Experiences, Integrated care, Multimorbidity, Registered nurse case manager, Person-centered care, Qualitative research

## Abstract

**Background:**

Multimorbidity is increasingly acknowledged as a significant health concern, particularly among older individuals. It is associated with a decline in quality of life and psychosocial well-being as well as an increased risk of being referred to multiple healthcare providers, including more frequent admissions to emergency departments. Person-centered care interventions tailored to individuals with multimorbidity have shown promising results in improving patient outcomes. Research is needed to explore how work practices within integrated care models are experienced from Registered Nurse Case Managers’ (RNCMs) perspective to identify areas of improvement. Therefore, the aim of this study was to describe RNCMs’ work experience with a person-centered collaborative healthcare model (PCCHCM).

**Methods:**

This study used an inductive design. The data were collected through individual interviews with 11 RNCMs and analyzed using qualitative content analysis.

**Results:**

Data analysis resulted in four generic categories: ‘Being a detective, ‘Being a mediator’, ‘Being a partner’, and ‘Being a facilitator of development’ which formed the basis of the main category ‘Tailoring healthcare, and social services to safeguard the patient’s best.’ The findings showed that RNCMs strive to investigate, identify, and assess older persons’ needs for coordinated care. They worked closely with patients and their relatives to engage them in informed decision-making and to implement those decisions in a personalized agreement that served as the foundation for the care and social services provided. Additionally, the RNCMs acted as facilitators of the development of the PCCHCM, improving collaboration with other healthcare professionals and enhancing the possibility of securing the best care for the patient.

**Conclusions:**

The results of this study demonstrated that RNCMs tailor healthcare and social services to provide care in various situations, adhering to person-centered care principles and continuity of care. The findings underline the importance of implementing integrated care models that consider the unique characteristics of each care context and adapt different case managers’ roles based on the patient’s individual needs as well as on the specific needs of the local setting. More research is needed from the patients’ and their relatives’ perspectives to deepen the understanding of the PCCHCM concerning its ability to provide involvement, security, and coordination of care.

**Supplementary Information:**

The online version contains supplementary material available at 10.1186/s12913-024-11500-3.

## Background

Globally, people are expected to live longer in the coming decades [1], and by 2030, one in six people will be aged 60 years or older. This age group is expected to double by 2050, reaching approximately 2.1 billion. In Sweden, the proportion of people aged 65 years and older is projected to increase to 23.5% by 2050 [2]. Individuals aged ≥ 70 years are increasingly in need of medical attention, with major health changes frequently occurring after the ages of 80–85 years [3]. Multimorbidity, defined as the coexistence of two or more chronic conditions, correlates with age. It affects 65% of people aged 65–84 and 82% of those aged 85 years and over [4 p. 3]. It is well known that people with multimorbidity have various chronic conditions, such as musculoskeletal disorders, mental health problems, and cardiovascular and metabolic diseases [4, 5]. Multimorbidity is linked to increased healthcare utilization, including more frequent admissions to emergency departments, longer hospital stays, functional decline, polypharmacy, poorer quality of life, decreased psychosocial well-being [4, 6], increased risk of being referred to other healthcare providers [7], and an increased financial burden on healthcare systems [4, 8].

Despite the high prevalence of multimorbidity in healthcare settings, clinical guidelines for the treatment and organization of healthcare often focus on single chronic conditions, and rarely on multiple conditions [5, 7]. There is often inadequate communication between healthcare providers regarding patients’ diverse needs, which adversely affects patients who have multimorbidity [6], which can result in these patients being left out of important clinical decisions [5]. Self-management ability is important for this vulnerable group and is affected by factors such as how the illness is perceived by the individual, their level of motivation, the strength and availability of their social networks, and the support options available to them [9]. Thus, it is important that care interventions for older persons with multimorbidity are person-centered.

Studies by Poitras et al. [10] and Søgaards et al. [11] indicated that person-centered care (PCC) interventions for individuals with multimorbidity could enhance patient outcomes. PCC emphasizes ethical, person-focused relationships, moving beyond viewing patients as a “passive target of a medical intervention” to involve the patients as an active and capable partner in the decision-making process of their own care [12 p.249, 13 p.1]. According to Ekman [13] ethics serve as the foundation for structuring person-centered care and this ethical perspective emphasize the importance of understanding the person behind the illness and actively involve them in their care. A central aspect is to establish a partnership between the patient and the healthcare professional, that builds on the patients’ personal narratives about their illness and its significant impact on their live. Establishing and working the partnership empowers and motivates patients to actively engage to their problems. From a registered nurse (RNs) perspective, person-centeredness involves establishing a genuine patient-nurse relationship, in which the person is the core of attention and employs a holistic perspective as a starting point in care [14]. Several stakeholders, such as the WHO [15], patient organizations [16], and healthcare professional organizations [17], have promoted the implementation of PCC in clinical practice.

Skou et al. [4] emphasized the importance of better integration of primary and secondary healthcare services and improved communication between care providers when providing care to patients with multimorbidity. A systematic review [18] showed that nurse-led interventions for patients with chronic conditions were associated with reduced hospital readmissions, decreased admissions to emergency departments and hospitalizations, improved continuity of care, and high levels of patient satisfaction. Furthermore, the care provided by an interprofessional team can help patients with multimorbidity address their complex needs [19]. In Sweden, regional and municipal autonomy have led to diverse care models nationally. Recently, the Swedish Association of Local Authorities and Regions [20] reported a nationwide shift toward PCC. Simultaneously, there is an increasing demand for collaborative care models that provide PCC [21]. Current work methods and structures are not designed to handle future demographic changes or the increasing need for healthcare among older persons with multimorbidity. In response, a person-centered collaborative healthcare model (PCCHCM) was developed to integrate healthcare for older persons with multimorbidity, thereby enhancing the quality of care. Further research is needed to explore how work practices within these integrated care models are experienced and to identify areas of improvement. Therefore, the aim of this study was to describe RNCMs’ work experience with a PCCHCM.

## Methods

### Design

This study employed a qualitative inductive design [22]. Semistructured interviews were used to collect the data, and inductive qualitative content analysis was conducted, as described by Elo and Kyngäs [23]. The study was conducted according to the Consolidated Criteria for Reporting Qualitative Research checklist [24].

### Setting

The Swedish healthcare system is primarily financed by tax revenue and consists mainly of publicly owned healthcare facilities, with only a limited portion privately owned [25]. The inhabitants can choose their primary healthcare provider. The healthcare system is decentralized and consists of 21 autonomous regions and 290 municipalities that provide healthcare regulated by the Health and Medical Services Act (2017:30) [26]. Primary Healthcare Centers (PHCs) are the foundation of Sweden’s healthcare system, offering care at the primary level, including medical treatment, prevention, nursing, and rehabilitation. Secondary healthcare, primarily hospital based, provides both inpatient and outpatient services. According to the Social Services Act (1980:620) [27] Swedish municipalities are mandated to provide care and housing for elderly people and disabled people. However, the coexistence of the Health and Medical Services Act (2017:30) [26], and the Social Services Act (1980:620) [27] risk leading to care fragmentation and difficulties accessing needed care, especially for those with multimorbidity who received care from multiple providers. Home healthcare services are also provided for older persons, ranging from basic to extensive services, and medical, rehabilitative, and nursing care, available either at their own homes or at specialized facilities, such as nursing or dementia care homes.

This study was conducted within a region with approximately 160,000 inhabitants, located in a sparsely populated area in southeast Sweden. This region includes five municipalities, one hospital located in two different cities, 18 PHCs providing healthcare services at the primary level to inhabitants, and 14 ambulances available in the region during day-evening time, of which seven were available around the clock. There are five home healthcare physicians available on call, one per municipality, working on behalf of PHCs and making emergency home visits to patient homes in all five municipalities. The PCCHCM was implemented in this region in June 2021 and has been continuously developed since then.

### The PCCHCM

The regional model, the PCCHCM, is based on cooperation and coordination between the regional healthcare authority (i.e., primary care, inpatient care, and prehospital care) and the municipal healthcare authority (i.e., social services and home care services). The goal is for the patient and their relatives to be involved, to experience increased security, and for the care provided to be coordinated, person-centered, and given at the right level of care. The coordination process, together with the advanced medical care plan, forms the basis of the model, along with the coordination functions that exist in both regional and municipal healthcare authorities. The coordination process should strengthen the proactive approach and early identification of older adults with multimorbidity and those at risk of developing frailty.

The coordination function is managed by Registered Nurses (RNs), who serve as case managers responsible for planning cohesive, seamless, and timely care in accordance with the patient’s needs. The coordination process involves identifying patients, assessing, and evaluating their needs, planning care and treatment, initiating and communicating interventions, and conducting follow-up. The PCCHCM also includes the possibility of initiating an advanced individual medical care plan for those identified as having multimorbidity. The plan is a structured documentation of the patient’s medical, nursing, and social status, including a detailed plan for patient care. The plan aims to guide healthcare professionals from both regional and municipal healthcare authorities in decision making regarding patient care when, for example, a deterioration in a person’s health condition occurs. Working in the PCCHCM also included evaluating and developing the model itself with involved healthcare professionals.

### Recruitment and participants

A purposive sampling strategy [22] was used to obtain a heterogeneous sample of participants recruited from both regional and municipal healthcare authorities, with variations in clinical experience and experience working with PCCHCM. Verbal information about this study was provided at several meetings with the RNCMs. If the RNCMs were interested in participating in the study, they were asked to contact the authors who provided detailed written and verbal information about the study and then booked time for the interview. A total of 22 participants were invited, 3 declined to participate (no reason was requested), and 8 did not contact the authors. The final sample consisted of 11 RNCMs (see Table 1).

**Table 1:** Characteristics of the participants (*n* = 11)

**Age (years)**		
Mean		43
Median		48
Range		29–64
**Sex (n)**		
	Female	10
	Male	1
**Professional education (n)**		
	RN^1^	11
Professional degree (n)	Diploma degree	0
Academic degree (n)	Bachelor degree	4
	Master degree	7
Specialist education (n)		7
**Clinical experience as RN** ^**1**^ **(years)**		
Mean		15
Median		15
Range		2–22
**Experience working within PCCHCM** ^**2**^ **(months)**		
Mean		15
Median		12
Range		3–28
**Employment (n)**		
Municipal healthcare authority		6
Regional healthcare authority		5

^1^ Registered Nurse

^2^ Person-centered collaborative healthcare model

### Data collection

The participants were offered the opportunity to choose between individual face-to-face interviews or video interviews via Microsoft Teams [28], as data collection took place during the post COVID-19 pandemic period (March – December 2023). All participants opted for individual video interviews, which were recorded on a dictaphone, transcribed verbatim, and checked for accuracy by two authors (MH and VU). The interview began with an overarching question: I am interested in your experiences working as an RNCM within the PCCHCM. Can you please tell me about your daily work. An interview guide containing open and probing questions was employed when needed if the informants’ answers to the overarching question did not cover the area of interest (see Appendix 1). A pilot interview was conducted to evaluate the validity of the overarching question and interview guide. As no amendments were necessary, this interview was included in this study. The interviews lasted 27 to 43 min (mean 36 min) and were pseudonymized to maintain the confidentiality of the participants. Each interview was assigned a number (1–11).

### Data analysis

The data were analyzed in accordance with Elo and Kyngäs’s [23] qualitative inductive content analysis method and started after all interviews were conducted. Qualitative content analysis is a research method using a systematic approach and is suitable to analyze interview texts with the aim to achieve a condensed and broad description of the phenomenon, resulting in categories describing the phenomenon. An inductive approach was chosen because of the limited prior knowledge of the phenomenon under investigation. Initially, multiple open-minded readings of the interview texts were conducted to become immersed in the data and to gain an overall understanding of the content. Second, the interview texts were transformed into codes by taking notes and creating headings at the margins of the interviews. Two authors (MH and EKA) independently performed this step. In the third step, the codes were organized into a coding sheet and grouped together based on similarities and differences, with a focus on the aim of the study. Two authors (MH and EKA) initially performed this step, and then discussed the codes and compiled them into a coding sheet. The fourth step involved interpreting similarities and differences to achieve further abstraction, resulting in four generic categories, including subcategories. Finally, the generic categories were grouped and reduced to the main category to represent the abstracted results. The first and last authors (MH and EKA) performed this analysis, while the other authors (AA and VU) validated it by reading the transcripts and actively participating in the discussions of the interpretation until consensus was reached. The original quotations from all participants were presented to illustrate the findings and the participants’ personal experience [29].

## Results

The RNCMs’ experience of their work in accordance with a PCCHCM were concluded in the main category, ‘Tailoring healthcare and social services to safeguard the patient’s best.’ The results indicate that the RNCMs strive to investigate, identify, and assess older persons’ needs for the coordination of care. Their endeavors involved receiving, prioritizing, and transmitting information and knowledge about patients’ needs and care plans to involved healthcare professionals. The RNCMs worked in partnership with both patients and their relatives to engage the patient in making informed decisions and implementing those decisions into a tailored agreement that became the foundation of the care and social services that the patient received. In their work, RNCMs acted as facilitators of the development of the PCCHCM, resulting in improving/enhancing collaboration with other healthcare professionals, thereby ensuring better possibilities for securing the patient’s best. In the subsequent results (see Table 2), the subcategories are interwoven in the description of the four generic categories.

**Table 2:** Overview of the findings; main category, generic categories, and subcategories

Main category:	Tailoring healthcare and social services for the patient’s best				
Generic categories:		**Being a detective**	**Being a mediator**	**Being a partner**	**Being a facilitator of development**
Subcategories:		Identifying patients at risk	Receiving, prioritizing, and transmitting information	Working in partnership with patients and relatives	Developing in collaboration
		Assessing patient’s needs	Mediating in a complex health system	Being representative of the patient	Performing regular work and evolving the PCCHCM^1^ simultaneously
		Shifting focus depending on context		Promoting continuity and security	Making the PCCHCM^1^ visible

^1^ Person-centered collaborative healthcare model

### Being a detective

The RNCMs experienced that their work involved detective activities in the sense of investigating, identifying, and assessing older persons with multimorbidity in need of coordination of care. The identification of patients who needed coordination of care could occur when other colleagues came up with inquiries both verbally and in writing. This could occur in collaboration with interprofessional teams or through patients or relatives contacting the RNCM themselves and asking for help. In inpatient care, RNs use screening with the Geriatric Rating Scale as a basis for further assessment of needed interventions. This screening can be performed by the RNCM in the geriatric department or by other healthcare professionals, for example, in ambulance services or emergency departments, who would then refer the patient to the RNCM. The primary goal of this detective work was to identify patients with multimorbidity in need of coordination of care. After the patients were identified, the RNCM assessed the interventions required by the patients. This could range from simple interventions, such as arranging a wheelchair, to more complex interventions, such as initiating collaboration with several stakeholders to plan how care and support at home can be offered to the patient.

“My daily work largely involves identifying these patients…, and I do that in collaboration with the healthcare professionals in the emergency department who screen. The ambulance also assists. Then, depending on the score that the patient receives on this screening tool, I reviewed the medical records to determine what kind of patient it is and what needs exist. Are there any gaps, could the care be better coordinated here… sometimes I bring up these patients for discussion, we have coordination meetings twice a week.” [Participant 8].

Being a detective was described as having an exploratory role in which the patient’s need for interventions was identified and assessed, and care was planned to achieve coordinated healthcare and social services. To be able to carry out interventions for the patients best, this usually happened in collaboration with various healthcare professionals from both regional and municipal healthcare authorities who were part of the PCCHCM (primary care, inpatient care, prehospital care, and home care). Common collaboration partners in RNCMs work included physicians, home health care physicians, RNs, physiotherapists, social workers, other RNCMs, occupational therapists, and others working within rehabilitation. Collaboration also occurred with the municipality’s social teams.

“You do this detective work together, then we go through the background again, we check the patient’s history, we look at the patient’s issues, what is causing them to be repeatedly admitted to the hospital, then you make a plan on what can be done moving forward, and how to keep this patient at home, and provide care at home without needing to send them to the hospital.” [Participant 1].

The focus of detective work could shift depending on what healthcare authorities the RNCM worked in. For example, in inpatient care, the role, to a greater degree, involved the identification and assessment of patients’ needs right now, while the RNCM also planned for the patient’s continued care at home, involving both primary care and/or home healthcare. In home healthcare, detective work, to a greater extent, was followed by planned interventions, ongoing assessments, and adapting interventions to patients’ changing needs. In primary care, detective work also involved identifying patients who were previously unknown to healthcare, and continuously assessing patients with multimorbidity whose care had already been coordinated. In all settings, the detective work involved identifying patients with acute conditions, for whom an assessment needed to be made on short notice.

“I receive messages from the hospital, from geriatrics, that this patient has been to the emergency department, this one needs help, they are admitted, they probably need a bit more help, we discuss what we can do, discuss with physicians, perform rounds with physicians and schedule them for visits.” [Participant 7].

### Being a mediator

RNCMs experienced that their work involved the responsibility of being mediators of information and knowledge about patients’ needs and care to other healthcare professionals. They compared their work to that of being the “spider in the web”. [Participant 11]. Being a mediator involved receiving and transmitting information, as well as prioritizing information in relation to patients’ needs for healthcare and social services. This meant involving other healthcare professionals, such as the home healthcare team to access this information. Common ways to convey information include written messages via digital platforms in the form of questions, reports, feedback, telephone contact, and various types of meetings with physicians and other healthcare professionals. In some care facilities, there is a special phone number to call the RNCM to facilitate collaborative efforts for the patient. RNCMs could also be contacted by the patient or their relatives and needed to mediate their information to the responsible healthcare professional or act on the given information themselves. In addition, being a mediator included documenting the patient’s individual care plan for continued care and distributing copies to both the patient and all those involved in the patient’s care.

“We work very closely with the home healthcare in (a name of a municipality), but I must say I think we have truly great collaboration. Many independent messages on digital platforms come from home healthcare; they range from physicians’ assessments, home visits, sampling, and medication renewals, so we are constantly sitting like a spider in this web.” [Participant 3].

Being a mediator also involved listening to the patient’s social needs and contributing to the patient’s social activities; for example, by mediating information to the patient about other services, such as the municipality’s day centers, libraries, or the church’s social activities. The RNCMs experienced that the contact pathways they had developed with other professionals worked well. As the PCCHCM evolved over time, the RNCMs became more familiar with each other’s work responsibilities, facilitating the mediation of patient information. However, mediator responsibility was hindered by the different medical records systems used by the healthcare authorities involved in patient care. This challenge was further compounded by the fact that RNCMs were also employed by different healthcare authorities, which sometimes complicated coordination and communication processes. The RNCMs worked under different legal frameworks, posing challenges in mediating and investigating the information that is important for patient care. One example is medication prescriptions that are difficult to review.

“We work in different medical records systems, so that is actually the biggest obstacle for collaboration… we have patients who go to the PHC themselves, we do not get any updates on medication adjustments, for example. If we do not get that information, the patient continues with the same prescriptions as before, so the medical records system is a big, it is an obstacle.” [Participant 2].

### Being a partner

RNCMs experienced that they strived to work in partnership with patients and their relatives. This included listening to and assessing the patient’s needs for healthcare and social services, as well as addressing their expectations and wishes. Being a partner involved providing information, engaging the patient in making informed decisions, and then implementing those decisions into a tailored agreement that became the foundation of the care the patient received. The RNCMs described that the partnership also involved the follow-up of the patient after they had been hospitalized, monitoring changes in medication prescriptions, conducting follow-up conversations in the patients’ homes, and, when necessary, making risk assessments. In the follow-up conversations, the RNCMs responded to questions from both patients and their relatives and guided them in the healthcare system, for example, regarding self-care. The partnership also entailed performing various assessments of the patient’s health status, both independently and on behalf of other healthcare professionals, and, based on this, planning, implementing, and evaluating different nursing interventions for the patient. This meant scheduling a visit to the geriatric nursing clinic as a follow-up after hospitalization or performing planned or urgent care interventions at the patient’s home. RNCMs expressed a desire to work closely with patients, which could involve making more comprehensive interventions in patients’ homes or working across departments within inpatient care to better meet the needs of older patients who require care coordination.

“I usually schedule telephone calls with them when they come home. And have follow-up conversations, and then it often emerges a lot when they come home. And they have questions, and some do not even know what happened in the hospital … It is this around-the-job work that I arrange. This collaboration with home healthcare, I arrange medication dispenser for them and self-care certificates and… and that is my daily work with coordination.” [Participant 5].

The RNCMs described that in the partnership, relatives played an important role in their extensive knowledge of the patient’s situation and needs, but it was also noted that their knowledge was not always sufficiently utilized by healthcare professionals. At the same time, the RNCMs expressed that some relatives’ interests were not always aligned with the patients’ wishes. For example, the patient might have wished to be cared for at home, but relatives preferred special accommodations. In such situations, RNCMs always represent the patient’s best interests.

The RNCMs described that the patient’s participation in the partnership was an important starting point, which included a person-centered approach in which they saw themselves as representatives of the patient. This meant supporting patients’ ability to take responsibility for their own care and supporting them in expressing themselves in what was most important to them. Being a representative of the patient also meant showing compassion for the patient, seeking feedback, and representing the patient’s best interests with various healthcare contacts. It also involved seeking alternative solutions and consulting with other healthcare professionals with the patient’s consent. To represent the patients’ best interests, the RNCMs needed to build a trust-filled relationship where the patients felt secure in expressing their wishes and trusted that they did not need to go to the emergency department to meet their care needs.

“Therefore, I try to get feedback from them on what we have discussed. Both that it is understood and that it is okay from their side as well. And during the conversation, you have to listen to what they want… there might be a patient who has a catheter who really does not want it… what possibilities there are to solve it in another way. Listen to the patient, be responsive, and try to see alternatives that might be better for them. Then, you might have to raise it with your physician. They might not be on that track but need to think a step further.” [Participant 6].

RNCMs had previous experiences with healthcare contacts with older patients, such as working in a geriatric nursing clinic or being responsible for the patient’s coordinated individual care plan. This previous work experience and relationships with the patients promoted continuity of care, as the patient had their RNCM to turn to, which in turn created continuity and safety in care. In their role as an RNCM, they identified patients whose medical needs required a coordinated advanced medical healthcare plan for their ongoing healthcare. They would then call for an interdisciplinary care meeting in which both the patients and their relatives participated.

“Advanced medical healthcare plan it is actually the very work tool that we have developed, where we have been able to see that yes, these care plans and coordinated individual care plans that are held, they are really important and good in every way but often there is maybe quite a lot of focus on Social Services Act interventions and on rehabilitation interventions, while the purely medical, I mean medical action plans and so on, get somewhat lost and thus we have developed this advanced medical healthcare plan, which is a tool to get a bit more of a holistic approach to the patient.” [Participant 10].

### Being a facilitator of development

The RNCMs experienced that their daily work also involved being facilitator of the development of the PCCHCM. This meant developing in collaboration, both within their own and with other professionals within the healthcare authorities involved in the PCCHCM, resulting in better possibilities of securing the patient’s best. Facilitation involved active participation in working groups with other RNCMs, operational managers, heads of care units, local authority senior medicine advisors, and the project leader, with the purpose of further developing the PCCHCM. The forms of the working groups varied, and digital meetings were considered beneficial for participation, as the participants were spread across different departments within both regional and municipal healthcare authorities. Physical meetings were seen as advantageous for getting to know each other and for facilitating collaboration in daily work. During these meetings, experiences were exchanged to improve working methods, processes, and routines. The interprofessional competencies of the RNCMs were developed as participants came from different healthcare authorities, which brought more perspective to the development of the PCCHCM. The RNCMs stated that from their collaborative work, they developed a structure in the form of routines, process descriptions, and direct contact routes. This structure facilitated the coordination of patient care and ensured that patients were cared for at the appropriate level.

“It (collaboration) has become much better, we collaborate with the municipality teams, both with the home visit team, safe discharge (intensive home care intervention), and the home healthcare team … we have rounds so the home healthcare team comes here, it’s a learning opportunity both for them and for us. The cooperation with the municipality teams is very good…. We call each other every week, me and the RNCM in the municipality, and check what kind of patients we have and how they are doing” [Participant 9].

The RNCMs described a desire to work more in teams with physicians to develop the PCCHCM. The lack of permanently employed physicians hindered this desire to collaborate and develop together. Instead, the RNCMs felt that they had to spend time supporting “hired physicians” who lacked knowledge about the local care context and patient familiarity. They expressed that physicians needed to be involved early in the process to make balanced decisions regarding the coordination of the patient’s healthcare and social services, as otherwise, they needed to make all decisions alone. Team collaboration has also been described as important for the sustainable use of resources. Furthermore, the RNCMs expressed a desire to systematically work with acute home visits performed within 24 h for patients with multimorbidity who had deteriorated to adjust their care plans in accordance with their actual needs. However, the shortage of physicians has made this difficult.

“I think a bit about working as an RNCM, especially at my PHC, it has been very difficult due to the shortage of physicians. This is an important aspect because elderly people with multimorbidity who primarily seek care, are shuffled by different physicians. Therefore, there is no continuity. However, they have no security. Thus far, there are only two permanent physicians.” [Participant 5].

Being a facilitator of development included evolving the PCCHCM and RNCMs’ roles while simultaneously performing regular work as RNs and RNCMs. The boundary between these commitments was often described as unclear, both for RNCMs and for the healthcare professionals around them. The understanding of the responsibility of RNCMs could be influenced by managers’ knowledge of the PCCHCM, which healthcare authority the RNCMs worked in, and a lack of knowledge about each other’s different areas of responsibility. Working as an RNCM was complicated by high healthcare professional turnover and a lack of geriatric competence. The RNCMs stated that their work had to be done within the existing time, which meant an increased workload that complicated participation in developing the RNCM’s role and providing care in accordance with the PCCHCM. The importance of allocating extra resources was highlighted; otherwise, collaboration according to the PCCHCM was at risk of not being prioritized. At the same time, the RNCMs described the significance of maintaining a positive outlook, exercising patience during the introductory phase, and being aware that development would take time.

The RNCMs described that the major development work ahead focused on making the PCCHCM more known to healthcare professionals in all involved organizations, as well as to patients and their relatives. If the PCCHCM is not sufficiently known, there is a risk that older persons with multimorbidity will not receive the coordination of care they need. There is a need for healthcare professionals to work together to develop a clear plan for communicating patient care with each other. This was considered urgent, as different traditions were experienced, where healthcare and social services had different focus areas, and this entailed a risk of misunderstandings in communication regarding patients in need of care coordination. The RNCMs described the need for a clear, simple pathway for patients who require coordination of care. Furthermore, the importance of preventive work has been emphasized. For example, in the form of expanding screening to identify additional at-risk patients in PHCs, which inpatient care to some extent has already been performed, or to develop a working method to plan for interventions at an early stage if the patient’s condition had deteriorated.

“We are invisible, people do not know we exist; not even the healthcare professionals in the ward know about us. Nor do those who might need help always know that we exist. It could be that healthcare professionals from regional healthcare authorities do not know how they should proceed for us to be involved. What is the procedure? Is it a referral procedure?” Is it enough to simply call. Or what to do to get us involved.” [Participant 4].

## Discussion

The main result of this study is presented in the main category: Tailoring healthcare and social services to safeguard the patient’s best, showing RNCMs’ sense of responsibility to purposefully contribute to the well-being of the patient. The findings highlight the importance of working in partnership and establishing trust-filled relationships with the patients. For RNCMs, this approach was required to safeguard the patient’s best interests, as the partnership facilitated their endeavor to identify, assess, and plan care interventions tailored to the patient’s individual needs. Similar findings have been reported from nurse case managers’ perspectives [30] and from patients’ perspectives [31], describing case managers serving as their advocates, standing on their side, and representing them in their various struggles with health and social care representatives. Our main result aligns with identified core components [32–33] when implementing PCC for older persons with multi-morbidity, e.g. knowing the older patient as a person, building trusting relationships, empowering the individual, and co-creating a tailored personal health plan. This indicates that the RNMCs worked in accordance with PCC principles to address the older person’s needs. In Sweden, there is an on-going national development towards providing accessible and integrated health and social care based on PCC, such as the PCCHCM. No research or evaluation related to PCCHCM has been previously published.

The results showed that the RNCMs strived to work in partnership with both patients and their relatives to create a tailored agreement based on shared decision-making, serving as the foundation of the care the patient received. Shared decision-making is one of the care processes in the person-centered practice emphasizing the therapeutic relationship’s role in recognizing patients’ beliefs and values during the decision-making process [34]. These findings align with Whitehead et al. [35], who, in a systematic review of qualitative studies, concluded that nursing care for persons with multimorbidity should integrate holistic assessment and PCC principles within an inter-professional and collaborative team framework. Research [36] has shown that RNs are healthcare professionals who are well-qualified to work with persons with multimorbidity, especially when care is based on PCC interventions that align with the needs of patients. However, the results showed that work of RNCMs was challenged by high healthcare professional turnover, a lack of geriatric competence, and the dual roles of working as an RN and an RNCM at the same time, which risk deprioritizing the work responsibilities of RNCMs.

From a nurse’s perspective [35], it has been shown that sufficient time is necessary to build an understanding of the patient’s condition, experience, and needs and to gain the patient’s trust in developing a therapeutic relationship necessary for care. Westlake et al. [37] concluded that the health professional’s role in supporting care partnerships and their knowledge of PCC is central to its practice. They advocated for increased education and training efforts in PCC to improve patient participation in healthcare. The results also showed that the partnership involved following the patients after they had been hospitalized, monitoring changes in their medication prescriptions, conducting follow-up conversations in the patients’ homes, and, when necessary, making risk assessments. Research from Sweden [38] reported that follow-up visits of patients after hospitalization performed by an RN allow monitoring of the patient’s health, which can lower the risk of deterioration and make the patient feel safer. Partnerships also involved creating trusting relationships between RNCMs and patients so that patients could feel safe expressing their care needs instead of going to the emergency department. Facchinetti et al. [39] reported that continuity of care interventions could prevent short-term hospital readmission in this patient group. Relationships are important for the development of partnerships. In a study by Scheffelaar et al. [40], the focus of care providers on individual patients was considered a core determinant of a good relationship. Thus, recognizing and understanding a patient’s individual needs and priorities are crucial for providing appropriate care interventions. To foster trusting relationships, continuity of care is a key and fundamental aspect of the quality of care [41]. Guthrie et al. [41 p.548] described relationship continuity as “Built on accumulated knowledge of patient preferences and circumstances that is rarely recorded in formal records and interpersonal trust based on experience of past care and positive expectations of future competence and care.” For RNCMs, this relationship continuity was important for representing the patient’s best interests in care situations.

The findings highlight that RNCMs need to adopt different roles to carry out their tasks in accordance with the PCCHCM. These roles were practiced in different ways depending on the organization in which they worked. Integrated and collaborative care models often introduce new forms of collaboration that can affect professional roles and responsibilities. A study by Gustafsson et al. [42] revealed that case managers experienced frustration due to unclear boundaries in their responsibilities, which impacted their work efficiency. According to SELFIE [43], a framework for integrated care for persons with multi-morbidity, it is important that the health care professionals, have clearly defined roles in how care is designed and coordinated. Having an assigned coordinator is considered particularly important, which also was included as a part of the RNMCs work activities. According to Joo and Huber [44], a mutual understanding of case management interventions is an important facilitator of its implementation. A common barrier described by both case managers and professional stakeholders is the lack of clarity regarding their roles. Complex care interventions, such as the PCCHCM, are in accordance with Torrey et al. [45] in need of clearly understood aims and roles; however, since such interventions are complex processes, it could be difficult to fully grasp the intervention. The SELFIE framework [43] underscores the importance of continuous professional education for RNCMs in integrated care for persons with multimorbidity. This education should encompass both soft skills, such as communication and teamwork, as well as managerial skills like case management. Additionally, supporting the development of new professional roles to address emerging demands is important.

One aspect of the PCCHCM, not commonly included in PCC interventions [11, 32], was the involvement of RNCMs in the development of the model itself, together with other healthcare professionals and managers. The findings presented in the sub-category ‘Being a facilitator of development’ highlight the importance of structured collaboration forums, such as working groups, for advancing the development of care pathways for practicing PCC. These collaborative efforts can also help identify and address the specific needs of the local context. Furthermore, the results indicated that RNCMs’ work responsibilities were understood differently by managers, depending on their knowledge of PCCHCM and a lack of knowledge about each other’s different areas of responsibility. Kirst et al. [46] conclude that integrated and collaborative care models must invest sufficient effort into building the necessary infrastructure to support trusting multidisciplinary team relationships, provider understanding of and commitment to the model.

Thus, introducing the PCCHCM, which is a complex care model involving both municipal and regional authorities, requires good planning for both joint work tasks and individual work that must occur within each organization. Martin et al. [47] concluded that open communication between case managers and leadership, and an improvement-focused culture, appear to be important elements of implementation success. Thus, a continuous evaluation system involving professional stakeholders and RNCMs could be used to achieve a shared understanding of the intervention, especially as the PCCHCM progresses over time. Wu [48] concluded that healthcare service delivery models must address the specific characteristics of different countries. According to Threapleton et al. [49], changes in integrated care interventions are more likely to succeed when local settings are well incorporated.

### Methodological considerations

In studies with qualitative designs, trustworthiness can be assessed by employing the concepts of credibility, confirmability, dependability, and transferability [50]. The study followed the guidelines outlined in the Consolidated Criteria for Reporting Qualitative Research [24]. The sampling strategy aimed to capture variation, resulting in a varied sample that included participants from both regional and municipal healthcare authorities with different ages, education levels, and lengths of experience working with PCCHCM. However, it is important to note that the participants were working within the PCCHCM in the same region, risking that their experiences might be colored by the organization’s structure and the culture of a specific region. The sample size of 11 RNCMs was small; however, the interviews revealed depth and richness in content contributing to the wide variation in the experiences of RNCMs necessary for the data analysis. One contributing factor to the sample size was the participants’ difficulty in participating due to their demanding work situation and staff shortage. This study included only one male RNCM, which could be considered a limitation. However, it is important to note that during the study period, almost all RNCMs working in the PCCHCM were female, which is common in the nursing profession. In Sweden, approximately 12% of all RNs are male.

The interviews were analyzed through ongoing collaboration and continuous discussions among the research team. This involved collaboration between the first and last authors within all steps of the analytical process, followed by the second and third authors reading all the data and having an ongoing discussion until consensus was reached, and four generic categories and the main category were formed. Two of the authors have clinical experience as RNs from inpatient care and home healthcare, and the third and fourth authors have experience working as research and development strategist/assistant with subject areas involving health and social care for older persons with multimorbidity. This preunderstanding can impact the interpretation of the data. However, efforts have been made to minimize such impacts by regularly moving back and forth between the interview data and the formed categories and maintaining a reflective and self-aware stance during the analytical process. Throughout the data analysis, critical reflections and regular discussions about the interpretations were conducted to ensure their trustworthiness. To ensure the confirmability of the results, quotations from all participants were provided to illustrate the findings and the participants’ personal experience. The results were presented in everyday language to remain close to the participants’ experiences. Efforts have been made to provide transparent descriptions of the study context, the participants’ characteristics, and the collection and analysis processes, giving the reader an opportunity to follow the different steps in the research process and facilitating reader assessment of transferable findings.

## Conclusions

The findings of this study provide insights into the RNCMs’ work experiences with a PCCHCM. These findings illustrate that RNCMs tailored healthcare and social services to safeguard patient’s best in different care situations, in line with PCC and with an emphasis on providing continuity of care. A supportive healthcare environment, including continuous education and ethical reflection, is necessary to promote RNCMs’ engagement in standing by the patient in partnership. Furthermore, the findings emphasize the need for integrated care models to address specific characteristics of each care context and to be flexible in the sense of adapting different case managers’ roles depending on the specific needs of the local setting and the patient’s individual needs. More research is needed from patients’ and their relatives’ perspectives to broaden the understanding of the PCCHCM concerning its ability to provide involvement, security, and coordination of care.

## Electronic supplementary material

Below is the link to the electronic supplementary material.

Supplementary Material 1

## Data Availability

The data generated during the current study are not publicly available, as the nature of qualitative data are difficult to fully anonymize. Participants in this study were not asked to agree that their data were publicly available. The datasets at an aggregated level in Swedish are available from the corresponding author upon reasonable request.

## Abbreviations

PCC: Person-centered care
PCCHCM: a Person-Centered Collaborative Healthcare Model
PHC: Primary Healthcare Center
RN: Registered Nurse
RNCM: Registered Nurse who serve as Case Manager
