# Supplementary material for: Registered nurse case managers’ work experiences with a person-centered collaborative healthcare model: an interview study
Source: BMC Health Serv Res. 2024 Sep 23;24:1108. doi: 10.1186/s12913-024-11500-3 (PMC11421112; doi:10.1186/s12913-024-11500-3)
Supplement: Supplementary file 1 — Supplementary Material 1 [file 12913_2024_11500_MOESM1_ESM.docx]

**Appendix 1**

**An interview guide**

The participant is provided with practical information on how the interview will proceed.

The interview begins with an overarching question: “I am interested in your experiences working as an RNCM within the PCCHCM. Can you please tell me about your daily work.”

**Possible follow-up questions that can be used when needed**

Please tell me about your role as an RNCM.

What expectations exists regarding your role?

Please tell me, what activities do you perform during your workday?

Please tell me, what are your experiences with the introductory phase of the PCCHM?

Is there anything else that you have not talked about regarding your work as an RNCM?

Please tell me, who do you collaborate with in your role as an RNCM?

Please tell me, in what ways do you collaborate with the older person, relatives, professionals or stakeholders in your role?

Please tell me, in what ways do you collaborate with other RNCMs?

What do you experience can promote or challenge collaboration within the PCCHM?

Please tell me, how do you, as an RNCM, work to involve the older person and relatives in PCCHM?

What do you experience can promote or challenge the participation of patients and their relatives in PCCHM?

What do you feel can promote or challenge the participation of professionals or stakeholders in the PCCHM?

Follow-up questions such as: Could you please give me an example?, Can you tell me more?, What do you mean? will be asked if needed throughout the interview to explore and deepen the topics that arise during the interview sessions.
